# Supplementary material for: Health service readiness to provide care for HIV and cardiovascular disease risk factors in low- and middle-income countries
Source: PLOS Glob Public Health. 2023 Sep 22;3(9):e0002373. doi: 10.1371/journal.pgph.0002373 (PMC10516419; doi:10.1371/journal.pgph.0002373)
Supplement: S3 Appendix — Comparison between CVDRF score derived using the SARA manual or the PEN guidelines. (DOCX) [file pgph.0002373.s003.docx]

# Appendix 3. Comparison between CVDRF score derived using the SARA manual or the PEN guidelines


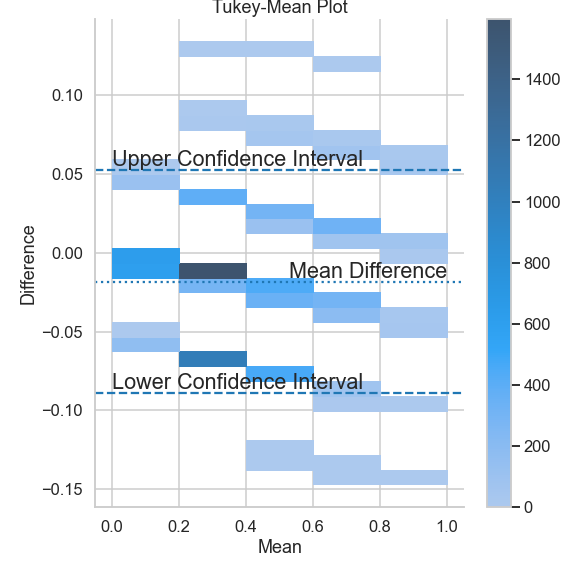


Mean Tukey histogram plot comparing CVDRF PEN and SARA scores at different ranges of the scores.

There were no substantial differences across readiness scores with only 292 of 7606 facilities falling outside 95% confidence intervals for differences between scores, suggesting that the two scores are reasonable approximations of each other for all levels of service readiness.

The 95% confidence interval for the Intra-Class Correlation coefficient for SARA and PEN CVDRF is greater than 0.98, and any value over 0.9 denotes excellent agreement between scores [47]. SARA and PEN scores are therefore taken to have excellent agreement, and so SARA CVDRF scores only have been used to compare against SARA HIV scores.
