# Supplementary material for: Health service readiness to provide care for HIV and cardiovascular disease risk factors in low- and middle-income countries
Source: PLOS Glob Public Health. 2023 Sep 22;3(9):e0002373. doi: 10.1371/journal.pgph.0002373 (PMC10516419; doi:10.1371/journal.pgph.0002373)
Supplement: Attachment — Submitted filename: Response to reviewers PLOS GH 30-06 v1.0 .docx [file pgph.0002373.s013.docx]

# Responses to the Editor

Thank you for your thorough read of our article. We have responded to your points below, and to peer reviewers further below. Editor and reviewer comments are in black, our responses in red and quoted amendments to our article are in italics.

1. We have noticed that you have uploaded Supporting Information files, but you have not included a list of legends. Please add a full list of legends for your Supporting Information files after the references list.

We apologise, this has been included with the resubmission.

**Additional Editor Comments (if provided):**

Overall:
1. The key overarching factors/categories influencing optimal healthcare provision include 1) governance i.e., government policies/strategies/guidelines, 2) healthcare system factors, 3) healthcare provider factors and 4) patient factors. These are multi-level, multiple and complex factors that interact and influence each other. Therefore, evaluating a single category is not necessarily a direct reflection of the ability to provide optimal care for a particular condition. The authors have failed to acknowledge these complexities in their paper.

Our response:

The editor raises an important comment on the complexity of health systems and healthcare provision beyond health service readiness. While our paper does focus on and present data for healthcare facility readiness factors, we acknowledge these complexities throughout the discussion such as stating that “These results concur with others’ findings that although facility readiness is a necessary component for ensuring [healthcare], it is not sufficient.”

To make this point clearer, we have added the following to the discussion:

*“Access to quality care leading to adequate treatment coverage/control goes beyond the availability of ready health services; these are necessary but not sufficient. Other elements of access to quality care are required to match service provision with outcomes. These include, but are not limited to, considerations of cultural norms, dimensions of geo-temporal access and affordability of services, the quality of the services provided, and individual and societal behavioural factors that affect care uptake, especially for chronic diseases.”*

We also discuss the role of governance and system factors in some detail.

2. Have SARA or SPA been validated? That is, found to be useful indicators of care for CVDRFs and HIV? What are the other factors that influence care that have not been considered?

Our response:

A systematic review of health service readiness tools considered SARA and SPA to be among the more reliable of the readiness assessment tools. <https://doi.org/10.1093/heapol/czu043> The questions asked on readiness are also backed up by observations, if appropriate, and data are quality checked. So, the surveys exhibit good construct validity for service readiness. In this regard, both SARA and SPA have been used to guide government policy in a wide range of LMICs. Whether the tools have good content or criterion validity for access to quality healthcare is questionable, but, access to quality healthcare would not be possible without the readiness of said healthcare. Access to quality healthcare is a highly complex issue, and dependent on multiple variables (e.g our cited article: Davies et al. <https://doi.org/10.1371/journal.pmed.1003268>).

3. Line 68: Please introduce the acronym LMICs here and not in line 75.

Our response:

Edited

4. Line 69: please be consistent with your use of introduced acronyms – CVD.

Our response:

Edited

5. Please pay close attention to acronyms throughout the paper e.g., line 85 – introduce acronym MDG before using it.

Our response:

Edited throughout

6. Lines 117-118: state clearly that this study focused on the 2012 surveys that were conducted in…list the countries.

Our response:

Apologies, this was an error which has been corrected.

7. Lines 134-140: The outcomes should be incorporated into the aims (currently vague) in lines 105-106. Do not present this section in point format.

Our response:

*“In this study, we aimed to determine readiness of health services to provide care for CVDRF across multiple countries by measuring a coverage score derived from SPA surveys.”*

*“We report 2 outcomes for all included facilities, which are the self-reported readiness of facilities to provide care and a facility coverage score of observed readiness for both CVDRF and HIV. We additionally report coverage scores in those facilities with self-reported readiness only.”*

8. It is unclear if the authors used SPA or SPA, and how does PEN fit in? Please explain clearly.

Our response:

Our response: we assume the editor means “SARA or SPA” and have responded with that consideration, below.

We used SPA data in our analysis, however, we note that SPA doesn’t have a derivation for CVD readiness. Therefore, given that SPA is based on SARA and shares multiple components, we use the SARA manual to construct our CVDRF readiness score.

However, the SARA coverage score has been criticised for missing certain components considered necessary for CVD risk reduction e.g. statins which should be prescribed for patients with diabetes. We therefore derived a second - more comprehensive - CVDRF readiness score containing care-elements detailed in the PEN guidelines. We used the SARA manual-derived CVDRF readiness score in our main analysis and that with additional PEN-derived variables in a sensitivity analysis. We found that the additional variables in our PEN-derived score did not affect the results. We have presented these findings in the appendix.

We have adjusted the manuscript as follows for lines 176-191:

*“A facility coverage score for CVDRF care (based on observed readiness) was constructed for all facilities based upon the presence or absence of up to 19 components required for CVDRF care as documented in the CVD and Diabetes sections of the SARA manual, given there is no score derivation for CVDRF from the SPA manual. Nevertheless, as SPA is derived from SARA and there are multiple shared components, the SARA manual can readily be used to derive readiness scores from SPA data. The coverage score was derived as the total number of care components for CVDRF that are present divided by the total if all components were present.*

*However, given that the SARA manual does not contain some CVDRF care-components considered essential in the World Health Organisation’s Package of Essential Non-Communicable Disease Intervention (PEN) guidelines, e.g: presence of statins in diabetes management. We conducted a sensitivity analysis using a second score derived from the practice recommendations contained in PEN.(World Health Organisation 2013) We used this score in a sensitivity analysis and appendix 2 contains a side-by-side comparison of components used to create readiness scores.”*

9. Line 141: What is considered a high score vs. a moderate or low score for the various outcome measures? These need to be clearly elucidated for the reader.

Our response:

We have avoided using absolute terms denoting high, low, and moderate, rather our intention is to compare and assess whether scores are relatively higher or lower than others. Please see also our response below to your comment number 12.

10. Line 201: The following is incorrect: ‘All variables are described as mean (%) when categorical…’ – means are not reported for categorical data.
Were the data skewed?

Our response:

This should have read ‘*All variables are described as frequency (%) when categorical…’* Continuous measures refer to the coverage score, which is a ratio measure from 0-1 and hence universally skewed.

11. Line 216: How many low-income and how many middle-income countries were included? Were there any differences between these 2 categories?

Our response

This has been added to the manuscript as follows:

“Eight countries (2 low and 6 middle income) representing a total of 13543 facilities…”

We have also identified individual country income status in Table 1.

12. What do the overall scores indicate in terms of readiness for provision of care in these 8 countries? Please provide the overall context before reporting on the comparisons.

Was coverage good or poor? Please describe the study findings using such phrases rather than simply presenting the data.

Our response:

Whilst we understand the desire to state categorically whether coverage is good or bad, we have preferred to use this analysis as a way of understanding what can be achieved, at best (for HIV), and compare that with other HIV or CVDRF scores. We also understand that multiple factors can be considered on the pathway to achieving, categorically, good or bad readiness. For example, health financing, disease burden, presence of advocacy groups etc. We therefore wish to avoid calling out countries for having bad or good readiness when we only have one – albeit important – element of health-system coverage that we are considering in this paper.

We also believe that it is not our place to judge what is categorically good or bad in the context of health systems in low resource settings which are attempting to provide services to their communities in often very trying situations. Indeed, whilst we could argue that 100% of availability of the essential elements for readiness outlined in the SPA survey is necessary to be “good”, no country achieved that in any subsample, even for HIV.

Rather, we wish to show what can be achieved by some countries and discuss the reasons why some countries may be doing better than others in order for them to learn. Thus, apart from when we have described that WHO has a target of 80% readiness for NCDs and that most facilities were below this, we prefer to not further judge as good or bad.

13. Lines 226-228: This belongs in the Methods.

Our response:

Edited

14. All tables should be standalone with acronyms explained in the text or footnotes – e.g., DRC, CVD/CVDRF

Our response:

Acronyms have been explained in all 3 tables.

15. The results are difficult to follow. Please consider presenting the overall key values/data in the text and summarising those for each country.

Our response:

We have adjusted the flow of the results to make reading easier. If the editor would additionally like us to present the values for all countries in the text and just describe results by further categories (individual countries, urban/rural etc) leaving the values for each category only in the tables, we can strip these out of the text.

16. Line 262: I could not find Fig 21. Please ensure correct labelling of all tables and figures.

Our response:

This has been edited and should read fig 2. Apologies for this error; while acknowledging this was our responsibility to correct during submission, we hope it could be helpful to point out there may be an error in the manuscript production software as none of the word documents submitted (or indeed created by us at any stage) appear to have this error in the text.

17. The Discussion is difficult to follow. The authors could perhaps make the text easier to follow by clearly describing what each outcome being discussed represents and summarising the key findings in the first paragraph.

Our response:

We have substantially edited the discussion and hope that it now flows better. We are very happy to provide further edits if necessary.

17. Lines 303-304: What were the coverage scores?

Our response:

We are not sure what the editor is requesting. We have followed scientific publication recommendations to not include results in the discussion, so have avoided responding by doing this and look forward to clarity.

18. Line 306: ‘21% greater’ than what? This sentence is unclear.

Our response:

Edited to read

*“median readiness score for CVDRF was substantially greater when analyses were restricted to facilities which reported providing services for CVDRF, compared with all facilities”*

19. Lines 319-321: Can the authors provide a reason/s for the low HIV coverage in Tanzania and Malawi? This would be of interest to the readers.

Our response:

We have added the following text:

*Figure 2 shows this is driven by lack of diagnostic testing in Tanzania, and by lack of staffing and information systems in Malawi.*

20. Line 360-361: please rephrase – what was controlled?

Our response:

Edited *“had their blood pressure well-controlled”*

21. Lien 363: What then is necessary? And so, what is the relevance of this study? And why is there a lack of concordance?

Our response:

As indicated in the responses to reviewers and as fully detailed in the rest of sentence, health facility readiness *is necessary, but not sufficient*. So, the question becomes, what *else* is necessary? It would not be possible to provide healthcare without a ready health facility (one simply cannot, for example, have HIV treated without medications). However, multiple other components are required to ensure that the patients who need to access those services indeed do access those services.

In the manuscript, we have added details as follows:

*“These results concur with Davies et al’s findings that although facility readiness is a necessary component for ensuring transit through the cascades, it is not sufficient. (10) Access to quality care leading to adequate treatment coverage/control goes beyond the availability of ready health services; these are necessary but not sufficient. Other elements of access to quality care are required to match service provision with outcomes. These include, but are not limited to, considerations of cultural norms, dimensions of geo-temporal access and affordability of services, the quality of the services provided, and individual and societal behavioural factors that affect care uptake, especially for chronic diseases.”*

Line 366: What is inconsistent? The authors need to improve their writing style and ensure clarity in their written text.

Our response:

Apologies for the lack of clarity.

This has been edited to: *Facility readiness may vary due to country level factors such as national policy, Direct Assistance for Health (DAH) funding, or national prevalence of diseases, although the relationships between these factors and readiness may vary by country, given the multiple other interacting factors involved in determining readiness.*

22. Line 374: ‘differences in facility readiness’ – what is the comparison here?

Edited *“Our measures of facility readiness should also be affected by disease epidemiology…”*

23. Line 379: Please include the reference for the prevalence. Are you comparing like with like? Please expand/explain. How reliable are the data i.e., death certification in these countries?

Our response:

Rates cited are for cause-specific incidence of death due to HIV or CVD, drawn from the Global Burden of Disease Study. Diabetes was not included in the comparison for simplicity. Although there is variation in death certification, the detailed methodology of the GBD “evaluates …data on incidence, prevalence, remission and mortality, for a disease, enforcing consistency between epidemiological parameters” (GBD 2019). While the methods will not account for all sources of bias, there are orders of magnitude of difference between HIV death rates between countries and these are highly unlikely to be due to bias in the methods of recording. These differences are smaller for CVD deaths but still suggests that healthcare needs as indicated by cause specific mortality rates do not explain differences in readiness scores.

The word “*prevalence”* at the beginning of the paragraph has been replaced with *“burden”* to more clearly reflect the comparison made.

24. Lines 382-383: Policies do not always translate into action i.e., implementation, and implementation is not necessarily effective, nor does it directly address what is needed. Please rework this section.

Our response:

In addition to edits in the preceding paragraphs which show the disconnect between suppose drivers of readiness and outcomes, we have edited this section to make it clearer, as follows:

*“Government policies and their implementation is also likely to influence facility readiness, but these alone are unlikely to lead to improved readiness. This is demonstrated by findings that NCD policy readiness scores reported by Allen et al*(Allen, Wigley, and Holmer 2021)*did not reflect CVD facility readiness scores that we saw in our study; for example the policy readiness score reported for Bangladesh was 38.9% and DRC 34.2%, where our median CVDRF readiness score in Bangladesh was 0.16 and in DRC was 0.36”.*

25. Lines 387-399: Some of this paragraph belongs at the beginning of the Discussion where the authors summarise their study findings.

Our response:

We have edited this accordingly along with substantial edits to the discussion to improve flow.

26. Line 423: please correct spelling. What respiratory diseases care are related to CVDRFs?

Our response:

We apologise, this was not our intended meaning. For clarity, we have edited this as follows:

*“We also used the question on self-reported facility readiness to provide NCD care as a marker of whether they provided CVDRF care, whereas facilities may have been responding positively to provision of care for respiratory diseases such as Chronic Obstructive Pulmonary Disorder rather than CVDRF”*

26. Lines 429 and 447 contradict each other in terms of representativeness. Please amend/clarify accordingly.

Our response:

We have clarified the first of the two lines to read:

*“However, other than Afghanistan, surveys were nationally representative, and Afghanistan was included as we believe it is still able to contribute valuable information of comparative facility readiness between HIV and CVDRF.”*

27. Lines 452-453: Briefly clarify/explain the Diabetes Compact (one sentence or phrase)

Our response:

Added context: *“The WHO’s ‘vision of reducing the risk of diabetes and ensuring that all people who are diagnosed with diabetes have access to equitable, comprehensive, affordable and quality treatment and care.’”*

28. Line 456: The conclusions should be an overall conclusion without referring to a single component, in this instance statins. Please rephrase. Further, is there testing of lipid profiles to warrant the stocking of statins? In low-income countries, ensuring access to adequate diabetes medicines is difficult enough, let alone focusing on statins. Was there sufficient access to diabetes and hypertension medications in the study countries?

Our response:

In line also with the reviewers’ comments, we have rephrased the conclusion as follows:

*“This study, which used a large, international dataset of nationally representative surveys with a stable inventory of questions over the study period, showed that the vast majority of health facilities surveyed in LMICs using the SPA tool are unprepared to deliver CVDRF services. It also indicated that sustained investment in improving care for chronic diseases, such as HIV, may be associated with improved readiness, but that even with this investment readiness remains suboptimal.*

*With increased focus on NCDs as part of the SDG agenda and through initiatives such as the Diabetes Compact,(Hunt et al. 2021) the WHO’s “vision of reducing the risk of diabetes, and ensuring that all people who are diagnosed with diabetes have access to equitable, comprehensive, affordable and quality treatment and care,” there may be substantially greater opportunities for policy makers to improve CVDRF care. Our findings provide policy makers, funders, and researchers evidence of where there are gaps in service provision which need to be filled to enable achievement of current global health goals.”*

# Responses to the Reviewers

## Reviewer 1

 I commend the purpose and premise of this analysis, however, although this involves a large data set, I don’t think this summary data answers the research purpose well, and the data has limited translatability.

Our response:

The intention of the analysis is to understand and compare service readiness for HIV and CVDRF. There are multiple potential methodologies that one could employ for this purpose. However, there are at current time, as far as we are aware, very few studies that have actually done this and no datasets other than the ones that we have used that allow these understandings to be generated, across countries. The SPA methodology is a widely used respected methodology to assess health service readiness in LMICs and is known to be rigorous and standardised (with some local deviations). Use of this data set therefore allows us to address our research question as rigorously as is currently possible.

Whilst it can be argued that all data collected in a certain well-defined areas has limited translatability, that these datasets are collected in multiple countries allows some understanding of the service readiness and comparison of this between HIV and CVRD. We have found that there are many findings which are shared across countries and therefore may be transferable elsewhere. We have also, in this discussion, attempted to discuss health system contexts of the included studies and understand what may be driving any differences seen. Nevertheless, we have included in the limitations a comment about the transportability of the findings:

*“The findings may not be transferable to all LMIC contexts; for example, only 2 low-income countries were included and no upper-middle income countries.”*

Comment

As outlined, the investment in HIV clinical research, HIV healthcare implementation and innovation has revolutionised the management of HIV enabling people living with HIV to live healthy, normal lives. Such global investment has not been observed for many other neglected communicable and non-communicable diseases (NCDs).
This research highlights the challenge of enabling better healthcare outcomes for non-communicable diseases (specifically cardiovascular disease and diabetes) associated with high morbidity and mortality, considering how we can leverage the achievements and integrate NCD/CVS care within HIV care in LMICs.

The article is written well, with clarity and with succinct descriptions.

Our response:

Thank you for this positive feedback

Cross-sectional data is taken from SPA surveys after 2012 (2014-2018) from eight countries and 7911 facilities.
The SPA tool is based on the WHO SARA tool, and includes additional questions to evaluate the quality of health services provided at health facilities. SPA assesses both the availability and quality of health services provided, including family planning, maternal and child health, HIV/AIDS, and other health services.
Overall, both SARA and SPA are useful instruments for assessing the readiness of health facilities to provide essential health services.

1) However, there are a few critical factors to consider when using these tools. For example, they rely heavily on the accuracy and completeness of data collected from health facilities, which can be challenging to obtain in some settings.

We agree that this is true for all surveys, but like most surveys that are considered reliable, delivering SPA entails thorough training of data collectors to minimise missingness and ensure high variable accuracy. The method used for the surveys also requires data collectors to directly observe the items in question and in most surveys 20% of facilities are also quality assured. Other authors using SPA surveys have observed <1% of data to be missing in facilities that participated in the survey (Lipira et al. 2018, reference 21).

Additionally, the indicators used to assess readiness may not be suitable for all settings and may need to be adapted to local contexts. Hence there is a need for caution,

All surveys were adapted to local contexts and guidelines, with items added or removed as appropriate. For example, the Bangladesh SPA survey included 3 different statins and 3 different sulfonylureas in their medications lists where most countries only considered 1 of each. We accepted the presence of any statin or sulfonylurea as the presence of the required item at a facility. Conversely, Afhganistan, Haiti and Senegal removed the question from their surveys on palliative care guidelines for HIV, and so we removed this from the denominator in calculating scores for these countries. Full details of how survey results were harmonised across countries are available in the data dictionary.

and it requires a combination with other data sources to provide a comprehensive assessment of health service delivery.

The reviewer rightly points out that readiness assessments do not provide a comprehensive assessment of delivery, and understanding availability, accessibility and utilisation of services is important. However, readiness of health facilities is a necessary pre-requisit to the provision of high quality health services and we belief that there is merit in this understanding. Please also see above comments to the Editor.

2) There is a large variation in the timing of data/results from countries (data from surveys completed in 2014 – 2018). Need to take into account the changes in services that may have occurred since surveys were completed, e.g >5 years ago.

It is an unfortunate issue that health facility readiness data are not regularly updated. We had included the most contemporaneous surveys that were conducted – within the past 10 years - and thus the results are as up to date as possible at the time of the analysis. Where longitudinal surveys are available, we analysed the second-most recent survey as a sensitivity analysis and found only slight increases in readiness but no significant changes in temporal trends between categories, countries and the two condition groups. Suggesting that health facility readiness is slow to change.. Furthermore, other authors (Kruk et al. 2016; Leslie, Spiegelman, et al. 2017; Moucheraud 2018) have had to use similarly historical data in their analyses. Finally, the comparison between two condition groups is an important element of this analysis and is not likely to be much affected by temporal changes. Nevertheless, we have added this as a limitation, as follows:

*“but selected that to be the most recent, to ensure results are as pertinent to current heath service readiness as possible, even though some of the surveys were nearly 10 years old.”*

*“These surveys took place between 2014-18 and so may no longer reflect current readiness or practice in HIV and CVDRF care.”*

3) From the tables – there is a significant variation in the ‘% of total surveyed’ across countries, for all three categories – Level of Care (primary/secondary), Management (private/public) and Geographic location (rural/urban)
Hence, interpretation of this data is limited due to the highly varied implementation of the WHO survey to assess readiness and service provision across primary and secondary care facilities across regions and across countries.
There is limited discussion about the potential bias related to why and which services would routinely and completely complete these surveys.

Our response

As indicated above, although the survey methodology is largely standardised, each country was free to implement the survey in a way which was thought to best show the readiness of the countries’ health system to provide care for the included conditions. Thus, apart from Afghanistan, all surveys are nationally representative using stratified sampling methodologies to ensure included facilities are representative of the health system. In addition, we show differences between facility types, level of care, or urban-rural setting. 96.5% of facilities completed surveys, and so missing data is minimal.

We have added the following to the limitations:

*Although the types of included facility vary by country, for all countries apart from Afghanistan, a stratified random sampling methodology was used to ensure representativeness of the countries health system.*

Given the vast differences/variations of contexts and health systems for regions, particularly across eight diverse countries, the translation of this data is limited.

Please see point above about transferability of findings. Additionally, a key finding is around the greater coverage of HIV items than CVDRF across most types of facilities; the consistency of this finding across contexts suggests that our findings may have transferability.

Translation of this data should be aligned with more robust service data that tailor surveys/investigations to the individual country and region contexts and health systems.
The tables clearly highlight this variation, although there is little content that highlights the vast differences in regions' contexts and health systems.

As discussed above, the surveys are tailored somewhat locally to ensure they are appropriate to the country under survey as well as remain comparable across countries. We also consider a range of differences between contexts in our discussion, although this is somewhat limited by the word count. For example, in paragraphs 6 & 7, we have considered burden of illness, direct assistance for health, healthcare outcomes and national policy implementation. While individually tailored investigations are very valuable, and several studies have analysed SPA results in individual countries, the value of our study is in making an international comparison using standards that can be applied effectively across the different contexts. In this way it is possible to see where findings are shared or diverge between settings and contexts, and which might be transferable elsewhere. The only way to do such comparisons in a way that is truly representative of all contexts is to carry out such surveys in all contexts. Unfortunately, as the limited number of SPA surveys conducted indicates, the resources are currently unlikely to be available for such a global assessment; we have used the most available data that there currently are to do this analysis.

The paper’s point about leveraging HIV healthcare system provision to manage CVS disease and diabetes is an important topic – although this data outlines a lack of preparedness to deliver CVDRF services and overall improved readiness to provide HIV services, much of this data may be outdated and lack representation. Additionally, it doesn’t assess how to bridge HIV healthcare system gaps and services for NCDs; and the ability to leverage HIV services.

Our response.

We have addressed many of these comments in our responses above. Our paper has highlighted that there is a possibility to leverage HIV care services to provide better CVDRF care, to assess how to do this would require multiple further studies and go beyond the scope of the paper. We have, however, brought into the discussion contemporary programmes such as the South Africa Ideal Clinics, which do attempt to bridge these gaps, as follows:

***“****Building on this idea, the Ideal Clinical program in South Africa plans for more integration of services. (45)”*

**Reviewer 2**

Thank-you for your positive comments, we have only addressed those which require action in our response.

Title(s)
Suggestion to make the running title read ‘Health service readiness to provide care for HIV and cardiovascular disease risk factors in low- and middle-income countries’

Our response:

We are happy with this suggestion and have adjusted the title accordingly.

Materials and Methods
- Line 190 onwards: Should it not read?’ Scores were created for six (instead of five stated here) domains …. categorized as amenities, equipment, diagnostics, information, medicines, and staffing, …..rather?

Our response:

Lines 215-217 have been edited, as follows:

*“Scores were created for the six domains of health service readiness described in the SARA manual,(World Health Organisation 2015) categorised as amenities, equipment, diagnostics, information, medicines, and staffing,* *although CVDRF scores contain no amenity domain and HIV scores contain no equipment domain”*

**Reviewer #3**: Overall comment
• It is a useful study. However, the main concern is old data from few countries. Another major concern is the lack of clarity as to how the program managers will use this information to improve the facility's readiness. In its current form, it is a highly theoretical academic exercise.

Our response: Thank you for the positive comment. We have addressed issues of data age and representativeness above.

Regarding the utility of the results to program managers’ ability to improve a facilities readiness. Many of the solutions to health service readiness require national policy development and implementation and financing. As reviewer 2 has commented, “*This is an important study. With its well acknowledged limitations, it would positively influence policies of Governments and Health facilities (in LMIC's), to improve CVD, CVDRF and HIV patient management and outcome.”* Our intent was that this article would be primarily impactful at a health system planning and financing level, including being of utility to donors who fund CVD and HIV programs, and additionally to researchers who need to understand where gaps in service readiness are to develop interventions to improve them. Only in providing this knowledge of lack of readiness can these actors being to address this.

We have addressed this in our conclusion, as follows:

*“With increased focus on NCDs as part of the SDG agenda and through initiatives such as the Diabetes Compact,(Hunt et al. 2021) the WHO’s “vision of reducing the risk of diabetes, and ensuring that all people who are diagnosed with diabetes have access to equitable, comprehensive, affordable and quality treatment and care,” there may be substantially greater opportunities for policy makers to improve CVDRF care”.*

Specific comments

Introduction
• The rationale for comparing HIV and CVD is not clear. These services are very different, and HIV programs have existed for much longer.

We argue in the paper that HIV and CVD are more similar than superficially would be expected, as both are chronic diseases which require long term follow up for populations. HIV programs have existed for longer and received much more attention, and hence there are lessons to be learned about the achievements in HIV that should be applied to CVDRF. In addition, we argue for a practical application of this analysis, such that HIV platforms and infrastructure could also deliver CVDRF care, which is likely to affect patients with HIV and which would be well suited to the long-term care offered by HIV services.

Methods
• Explain the key variables included in the five domains mentioned in Figure 2.

Our response:

We are unclear if the reviewer wished us to explain why these variables have been chosen or to describe them. Regarding the former, these variables were selected for inclusion during development of the USAID Demographic and Health Survey (DHS) SPA surveys as they reflected health service readiness to provide essential services. Regarding the latter, a full data dictionary is available in the appendix. In total there are 39 components in the 5 domains; including all in the main text may leave the main text bloated, and so we have placed them in the data dictionary where they can be reviewed more easily. If the editor would like us to add these to the main text, we are happy to do so.

• Define what is included in CVDRF services.

Our response:

This is included in the methods section of the paper, as follows:

‘Facilities were considered to have facility-reported readiness to provide services for CVDRF care if they responded positively to a general question asking about the provision of services as follows: “Does this facility offer any of the following client services? In other words, is there any location in this facility where clients can receive any of the following services…Diagnosis or management of non-communicable diseases, specifically diabetes, cardiovascular diseases, and chronic respiratory conditions in adults.” ’

“A facility score for CVDRF care (based on observed readiness) was derived for all facilities based upon the presence or absence of up to 19 components required for CVDRF care as documented in the CVD and Diabetes sections of the SARA manual. The SARA manual was used given there is no score for CVDRF from the SPA manual. Nevertheless, as SPA is based upon SARA and there are multiple shared components, the SARA manual can readily be used to derive readiness scores from SPA data. This score was derived as the total number of care components for CVDRF that are present in a facility divided by the total if all components were present. “

Results
• Table 2 – N for facilities included in the “coverage score” and facilities included in the analysis for “Coverage scores in facilities with self-reported” should be mentioned before the mean score.

We have considered carefully how best to present the results section and although we agree with the reviewer that n would be relevant, we would like to avoid adding more data into the text and making it harder to follow. Frequencies are presented in table 2 and the reader are likely to find it easier to read these from the table than from the text; hence we have not repeated n in the text.

The analysis reports median scores, rather than mean scores.

• Was the availability of drugs/diagnostics included in the scores for the private sector? This may not be relevant as most private doctors only give prescriptions to buy drugs from pharmacies/labs.

Our response

This point is well taken. However there is no clear observation that privately managed facilities have fewer drugs available than other sector facilities. For example, for CVDRF drugs, the private sector has greater coverage, while for HIV, the public sector has greater coverage. The surveys investigate facilities rather than individual clinicians; given this, even private facilities are likely to have dispensaries to hand.

• Present more specific information about the gaps in each of the domains (at least a few) to make the data useful for program managers.

Our response:

Appendix 8 provides this information in greater detail for all domains and individual items and is signposted in line 281; we had erroneously not submitted a table of legends in our submission and so this will be more clearly signposted in our resubmission.

Discussion

• Due to the complexity and length of data collection tools, these survey tools may lead to poor-quality data in low-resource settings. Authors should reflect on how these surveys can be made more useful and practical based on their analysis. Are there any specific suggestions about changing/modifying some of the questions to make them more useful for analysis and interpretation?

Our response:

Whilst the point about low quality data is noted, as addressed above, the data collectors for SPA do undergo rigorous training. Also may of their inputs are quality-checked. However, we note three specific points about data collection for facility readiness; the first two pertain to previous comments on representativeness and transferability. Given the importance of health service readiness to achieving the Sustainable Development Goals, we urge first that these surveys are updated more regularly; second that their geographical coverage is extended; and third, that similar SARA surveys from held by WHO are made available to increase the usability of these surveys for cross national learning.

• The authors have highlighted the disconnect between scores and treatment coverage/control in the discussion. It further raises the question about the usefulness of these surveys. It is important to reflect if the questions truly capture what is useful. Were there variables which were not useful and could be removed? Were there variables which could not be appropriately interpreted? For example, the issue of adequacy of medications which will depend on the patient load, may not answer the issue of adequacy of medications. If the information systems are weak, this information may not be available, hence limiting the usefulness of drug data.

Our response:

In general, we found the tools well designed with clear methodologies and rationales, low missingness, and with rigorous implementation guidance and details. We also maintain that health service readiness – which we have assessed – is a key prerequisite to obtaining quality health outcomes, as well documented by Donabedian (Donabedian 1988), in the WHO Building Blocks (World Health Organization. 2010), and most recently by Kruk et al.(Kruk et al. 2018) However, access to quality care leading to adequate treatment coverage/control goes beyond the availability of ready health services; these are necessary but not sufficient. Other elements of access to quality care are required to match service provision with outcomes. These include, but are not limited to, considerations of cultural norms, dimensions of geo-temporal access and affordability of services, the quality of the services provided, and individual and societal behavioural factors that affect care uptake, especially for chronic diseases.

We have now made this more explicit in our discussion, as follows:

*Access to quality care leading to adequate treatment coverage/control goes beyond the availability of ready health services; these are necessary but not sufficient. Other elements of access to quality care are required to match service provision with outcomes. These include, but are not limited to, considerations of cultural norms, dimensions of geo-temporal access and affordability of services, the quality of the services provided, and individual and societal behavioural factors that affect care uptake, especially for chronic diseases.*

**Reviewer #4**: On whether the statistical analysis has been performed appropriately and rigorously;
The methodology has not factored in the varying disease burden for both HIV and NCD for the different countries, nor adjusted for the same in the statistical analysis.

Our response:

Our intention was to understand the readiness of services and whilst we acknowledge that a determinant of readiness should be disease burden, we note that it is not always such a neat relationship. Many LMICs develop services, not based around disease burden, but rather around funding available, especially when that funding is from development assistance for health which is often earmarked for the specific diseases the funder has an interest in. This is well discussed in Dieleman et al (Dieleman et al. 2017) Additionally, the effects of increased disease burden could both increase or decrease expected readiness. To thoroughly understand if readiness matches diseases burden would require a further analysis accounting for relative prevalence diseases and other factors such as the broader health system or economic environment. This is an interesting question for a future analysis, but goes beyond the scope of this paper.

In analyzing the scores, whether primary, secondary or other; rural versus urban, private versus public, the author is not clear on whether these facilities are matched based on a clear criteria for comparison across the different countries

Our response:

These classifications were largely produced by national ministries of health and drawn from their master lists of facilities. As noted by other authors (McBride and Moucheraud 2022) there is no universal classification of urban vs rural; conversely, private vs public management is clearer and reported in national accounts (WHO 2017). Primary and secondary care facilities are reported in detail in the data dictionary.

Beyond restricting analysis to only facilities with self-reported service, the author could consider restricting to those with coverage scores as well in looking at access and management of NCDs to further support their conclusion.

Our response:

We are sorry, but we are unsure what the reviewer is asking for. All included facilities have coverage scores.

**Reviewer #5**: Over the years communicable diseases have been prioritised over non-communicable diseases, the leading cause of morbidity and mortality worldwide especially, in low and middle income countries, making the research topic critical to global health. Overall, I would recommend this paper for publication with a minor revision.

Our response:

Thank-you for your positive comment

The author gives a decent description of the research method. However I would recommend highlighting evidence to support the effectiveness and suitablity of the adopted method to the research topic. The limitations of the methods, I believe is worth mentioning here. Which of the services that inform readiness are operational or only available? Are there any bias?

Our response:

We had added a note to the limitations to indicate that reported service readiness doesn’t mean that it is always available or that there is equitable access to that service, as follows:.

*“This study is not able to directly link readiness to receipt of high quality care, and there may be unmeasured variation in the ability of different countries to convert the necessary but not sufficient readiness into high quality care”.*

We have addressed other issues of methodology in responses to other reviewers, above. We also note that although the methodology has limitations, other authors have used it to compare and assess health systems for other conditions (Kruk et al. 2016; Leslie, Spiegelman, et al. 2017; Leslie, Sun, and Kruk 2017; Moucheraud 2018). A systematic review of readiness tools by Nickerson et al. (Nickerson et al. 2015) found that SARA (the tool we used to produce the readiness score) considered both non-communicable diseases and HIV. While they criticised the lack of standardisation between data collection tools, they are not able to identify a standard a tool should meet or to assess the quality of individual tools.

Great use of heatmaps in results presentation. Generally good comparisons drawn in relation to public/private and rural/urban facilities.

Our response:

Thank-you for your positive comment

In concluding, the author backs a statement (not supported by evidence) with cost-effectiveness of managing CVDRFs which could be misleading. Is this with respect to HIV here? Is this just a thought? This should be clear as even not all CVD interventions are that cost effective.

Our response:

We agree that statement was misleading and have removed it from the conclusion.

# References

Allen, Luke N., Simon Wigley, and Hampus Holmer. 2021. “Implementation of Non-Communicable Disease Policies from 2015 to 2020: A Geopolitical Analysis of 194 Countries.” *The Lancet Global Health* 9(11):e1528–38. doi: 10.1016/S2214-109X(21)00359-4/ATTACHMENT/BCB612D0-47C3-421F-A11B-A0466F14862B/MMC1.PDF.

Anon. 2014. *MALAWI Service Provision Assessment (SPA) 2013-14*. Lilongwe, Malawi.

Bintabara, Deogratius, Alex Ernest, and Bonaventura Mpondo. 2019. “Health Facility Service Availability and Readiness to Provide Basic Emergency Obstetric and Newborn Care in a Low-Resource Setting: Evidence from a Tanzania National Survey.” *BMJ Open* 9(2). doi: 10.1136/bmjopen-2017-020608.

Dieleman, Joseph, Madeline Campbell, Abigail Chapin, Erika Eldrenkamp, Victoria Y. Fan, Annie Haakenstad, Jennifer Kates, Yingying Liu, Taylor Matyasz, Angela Micah, Alex Reynolds, Nafis Sadat, Matthew T. Schneider, Reed Sorensen, Tim Evans, David Evans, Christoph Kurowski, Ajay Tandon, Kaja M. Abbas, Semaw Ferede Abera, Aliasghar Ahmad Kiadaliri, Kedir Yimam Ahmed, Muktar Beshir Ahmed, Khurshid Alam, Reza Alizadeh-Navaei, Ala’a Alkerwi, Erfan Amini, Walid Ammar, Stephen Marc Amrock, Carl Abelardo T. Antonio, Tesfay Mehari Atey, Leticia Avila-Burgos, Ashish Awasthi, Aleksandra Barac, Oscar Alberto Bernal, Addisu Shunu Beyene, Tariku Jibat Beyene, Charles Birungi, Habtamu Mellie Bizuayehu, Nicholas J. K. Breitborde, Lucero Cahuana-Hurtado, Ruben Estanislao Castro, Ferran Catalá-López, Koustuv Dalal, Lalit Dandona, Rakhi Dandona, Pieter De Jager, Samath D. Dharmaratne, Manisha Dubey, Carla Sofia E. Sa Farinha, Andre Faro, Andrea B. Feigl, Florian Fischer, Joseph Robert Anderson Fitchett, Nataliya Foigt, Ababi Zergaw Giref, Rahul Gupta, Samer Hamidi, Hilda L. Harb, Simon I. Hay, Delia Hendrie, Masako Horino, Mikk Jürisson, Mihajlo B. Jakovljevic, Mehdi Javanbakht, Denny John, Jost B. Jonas, Seyed M. Karimi, Young Ho Khang, Jagdish Khubchandani, Yun Jin Kim, Jonas M. Kinge, Kristopher J. Krohn, G. Anil Kumar, Hassan Magdy Abd El Razek, Mohammed Magdy Abd El Razek, Azeem Majeed, Reza Malekzadeh, Felix Masiye, Toni Meier, Atte Meretoja, Ted R. Miller, Erkin M. Mirrakhimov, Shafiu Mohammed, Vinay Nangia, Stefano Olgiati, Abdalla Sidahmed Osman, Mayowa O. Owolabi, Tejas Patel, Angel J. Paternina Caicedo, David M. Pereira, Julian Perelman, Suzanne Polinder, Anwar Rafay, Vafa Rahimi-Movaghar, Rajesh Kumar Rai, Usha Ram, Chhabi Lal Ranabhat, Hirbo Shore Roba, Joseph Salama, Miloje Savic, Sadaf G. Sepanlou, Mark G. Shrime, Roberto Tchio Talongwa, Braden J. Te Ao, Fabrizio Tediosi, Azeb Gebresilassie Tesema, Alan J. Thomson, Ruoyan Tobe-Gai, Roman Topor-Madry, Eduardo A. Undurraga, Tommi Vasankari, Francesco S. Violante, Andrea Werdecker, Tissa Wijeratne, Gelin Xu, Naohiro Yonemoto, Mustafa Z. Younis, Chuanhua Yu, Zoubida Zaidi, Maysaa El Sayed Zaki, and Christopher J. L. Murray. 2017. “Evolution and Patterns of Global Health Financing 1995-2014: Development Assistance for Health, and Government, Prepaid Private, and out-of-Pocket Health Spending in 184 Countries.” *The Lancet* 389(10083):1981–2004. doi: 10.1016/S0140-6736(17)30874-7.

Donabedian, Avedis. 1988. “The Quality of Care.” *JAMA* 260(12):1743. doi: 10.1001/jama.1988.03410120089033.

Hunt, Daniel, Bianca Hemmingsen, Alena Matzke, Cherian Varghese, Asmus Hammerich, Silvana Luciani, Anselm Hennis, Francesco Branca, Fiona Bull, Nino Berdzuli, Jean Marie Dangou, Razia Pendse, Angela Pratt, and Bente Mikkelsen. 2021. “The WHO Global Diabetes Compact: A New Initiative to Support People Living with Diabetes.” *The Lancet Diabetes and Endocrinology* 9(6):325–27.

Kruk, Margaret E., Anna D. Gage, Catherine Arsenault, Keely Jordan, Hannah H. Leslie, Sanam Roder-DeWan, Olusoji Adeyi, Pierre Barker, Bernadette Daelmans, Svetlana V. Doubova, Mike English, Ezequiel García Elorrio, Frederico Guanais, Oye Gureje, Lisa R. Hirschhorn, Lixin Jiang, Edward Kelley, Ephrem Tekle Lemango, Jerker Liljestrand, Address Malata, Tanya Marchant, Malebona Precious Matsoso, John G. Meara, Manoj Mohanan, Youssoupha Ndiaye, Ole F. Norheim, K. Srinath Reddy, Alexander K. Rowe, Joshua A. Salomon, Gagan Thapa, Nana A. Y. Twum-Danso, and Muhammad Pate. 2018. “High-Quality Health Systems in the Sustainable Development Goals Era: Time for a Revolution.” *The Lancet Global Health* 6(11):e1196–1252.

Kruk, Margaret E., Hannah H. Leslie, Stéphane Verguet, Godfrey M. Mbaruku, Richard M. K. Adanu, and Ana Langer. 2016. “Quality of Basic Maternal Care Functions in Health Facilities of Five African Countries: An Analysis of National Health System Surveys.” *The Lancet Global Health* 4(11):e845–55. doi: 10.1016/S2214-109X(16)30180-2.

Leslie, Hannah H., Donna Spiegelman, Xin Zhoub, and Margaret E. Kruka. 2017. “Service Readiness of Health Facilities in Bangladesh, Haiti, Kenya, Malawi, Namibia, Nepal, Rwanda, Senegal, Uganda and the United Republic of Tanzania.” *Bulletin of the World Health Organization* 95(11):738–48. doi: 10.2471/BLT.17.191916.

Leslie, Hannah H., Zeye Sun, and Margaret E. Kruk. 2017. “Association between Infrastructure and Observed Quality of Care in 4 Healthcare Services: A Cross-Sectional Study of 4,300 Facilities in 8 Countries.” *PLoS Medicine* 14(12). doi: 10.1371/journal.pmed.1002464.

Leslie, Hannah H., Xin Zhou, Donna Spiegelman, and Margaret E. Kruk. 2018. “Health System Measurement: Harnessing Machine Learning to Advance Global Health.” *PLOS ONE* 13(10):e0204958. doi: 10.1371/JOURNAL.PONE.0204958.

McBride, Kaitlyn, and Corrina Moucheraud. 2022. “Rural-Urban Differences: Using Finer Geographic Classifications to Reevaluate Distance and Choice of Health Services in Malawi.” *Health Systems and Reform* 8(1). doi: 10.1080/23288604.2022.2051229.

Moucheraud, Corrina. 2018. “Service Readiness for Noncommunicable Diseases Was Low in Five Countries in 2013-15.” *Health Affairs* 37(8):1321–30. doi: 10.1377/hlthaff.2018.0151.

Nickerson, Jason W., Orvill Adams, Amir Attaran, Janet Hatcher-Roberts, and Peter Tugwell. 2015. “Monitoring the Ability to Deliver Care in Low- and Middle-Income Countries: A Systematic Review of Health Facility Assessment Tools.” *Health Policy and Planning* 30(5):675–86. doi: 10.1093/HEAPOL/CZU043.

Seiglie, Jacqueline A., Edson Serván-Mori, Tahmina Begum, James B. Meigs, Deborah J. Wexler, and Veronika J. Wirtz. 2020. “Predictors of Health Facility Readiness for Diabetes Service Delivery in Low- and Middle-Income Countries: The Case of Bangladesh.” *Diabetes Research and Clinical Practice* 169. doi: 10.1016/j.diabres.2020.108417.

World Health Organisation. 2013. *Implementation Tools Package of Essential Noncommunicable (PEN) Disease Interventions for Primary Health Care in Low-Resource Settings*. Luxembourg.

World Health Organisation. 2015. *HEALTH FACILITY ASSESSMENT OF SERVICE AVAILABILITY AND READINESS Service Availability and Readiness Assessment (SARA) An Annual Monitoring System for Service Delivery Reference Manual*.

World Health Organization. 2010. *Monitoring the Building Blocks of Health Systems : A Handbook of Indicators and Their Measurement Strategies*. World Health Organization.
